# Natural flavonoid glycosides Chrysosplenosides I & A rejuvenate intestinal stem cell aging via activation of PPARγ signaling

**DOI:** 10.1093/lifemedi/lnae025

**Published:** 2024-06-28

**Authors:** Jinbao Ye, La Yan, Yu Yuan, Fang Fu, Lu Yuan, Xinxin Fan, Juanyu Zhou, Yuedan Zhu, Xingzhu Liu, Gang Ren, Haiyang Chen

**Affiliations:** Laboratory of Stem cell and anti-Aging Research, Frontiers Science Center for Disease-related Molecular Network, State Key Laboratory of Respiratory Health and Multimorbidity and National Clinical Research Center for Geriatrics, West China Hospital, Sichuan University, Chengdu 610041, China; Laboratory of Stem cell and anti-Aging Research, Frontiers Science Center for Disease-related Molecular Network, State Key Laboratory of Respiratory Health and Multimorbidity and National Clinical Research Center for Geriatrics, West China Hospital, Sichuan University, Chengdu 610041, China; Laboratory of Stem cell and anti-Aging Research, Frontiers Science Center for Disease-related Molecular Network, State Key Laboratory of Respiratory Health and Multimorbidity and National Clinical Research Center for Geriatrics, West China Hospital, Sichuan University, Chengdu 610041, China; Laboratory of Stem cell and anti-Aging Research, Frontiers Science Center for Disease-related Molecular Network, State Key Laboratory of Respiratory Health and Multimorbidity and National Clinical Research Center for Geriatrics, West China Hospital, Sichuan University, Chengdu 610041, China; Research Center of Natural Resources of Chinese Medicinal Materials and Ethnic Medicine, Jiangxi University of Chinese Medicine, Nanchang 330004, China; Laboratory of Stem cell and anti-Aging Research, Frontiers Science Center for Disease-related Molecular Network, State Key Laboratory of Respiratory Health and Multimorbidity and National Clinical Research Center for Geriatrics, West China Hospital, Sichuan University, Chengdu 610041, China; Laboratory of Stem cell and anti-Aging Research, Frontiers Science Center for Disease-related Molecular Network, State Key Laboratory of Respiratory Health and Multimorbidity and National Clinical Research Center for Geriatrics, West China Hospital, Sichuan University, Chengdu 610041, China; Laboratory of Stem cell and anti-Aging Research, Frontiers Science Center for Disease-related Molecular Network, State Key Laboratory of Respiratory Health and Multimorbidity and National Clinical Research Center for Geriatrics, West China Hospital, Sichuan University, Chengdu 610041, China; Laboratory of Stem cell and anti-Aging Research, Frontiers Science Center for Disease-related Molecular Network, State Key Laboratory of Respiratory Health and Multimorbidity and National Clinical Research Center for Geriatrics, West China Hospital, Sichuan University, Chengdu 610041, China; Research Center of Natural Resources of Chinese Medicinal Materials and Ethnic Medicine, Jiangxi University of Chinese Medicine, Nanchang 330004, China; Laboratory of Stem cell and anti-Aging Research, Frontiers Science Center for Disease-related Molecular Network, State Key Laboratory of Respiratory Health and Multimorbidity and National Clinical Research Center for Geriatrics, West China Hospital, Sichuan University, Chengdu 610041, China

**Keywords:** intestinal stem cell, aging, chrysosplenoside, *Drosophila*, PPAR

## Abstract

The decline in intestinal stem cell (ISC) function is a hallmark of aging, contributing to compromised intestinal regeneration and increased incidence of age-associated diseases. Novel therapeutic agents that can rejuvenate aged ISCs are of paramount importance for extending healthspan. Here, we report on the discovery of Chrysosplenosides I and A (CAs 1 & 2), flavonol glycosides from the Xizang medicinal plant *Chrysosplenium axillare* Maxim., which exhibit potent anti-aging effects on ISCs. Our research, using *Drosophila* models, reveals that CAs 1 & 2 treatments not only restrain excessive ISC proliferation, thereby preserving intestinal homeostasis, but also extend the lifespan of aging *Drosophila*. In aged mouse intestinal organoids, CAs 1 & 2 enhance the growth and budding of intestinal organoids, indicating improved regenerative capacity. Mechanistic investigations show that CAs 1 & 2 exert their effects by activating the peroxisome proliferator-activated receptor-gamma (PPARγ) and concurrently inhibiting the epidermal growth factor receptor (EGFR) signaling pathways. Our findings position CAs 1 & 2 as promising candidates for ameliorating ISC aging and suggest that targeting PPARγ, in particular, may offer a therapeutic strategy to counteract age-related intestinal dysfunction.

## Introduction

Aging is a complex process that impairs the regenerative potential of adult stem cells, particularly in tissues with high cellular turnover such as the gastrointestinal tract [1–3]. The decline in intestinal stem cell (ISC) function with age is a critical factor contributing to a spectrum of age-related pathologies, including but not limited to atrophy, inflammatory bowel disease, and gastrointestinal cancers [4, 5]. Enhancing our understanding of the mechanisms underlying ISC aging and developing interventions to combat this decline is of utmost importance for promoting gastrointestinal health and longevity.

Recent research has highlighted the anti-aging potential of natural substances, especially flavonoids, which are abundant in fruits and vegetables [6, 7]. These compounds, including anthocyanins, procyanidin C1, baicalin, and quercetin, have garnered attention for their low toxicity and potential to mitigate aging and its associated diseases [7–9]. Our research has previously found that quercetin plays an important role in delaying the aging of adult stem cells [9]. Despite the promising nature of these discoveries, research focusing on the rejuvenation of ISCs to improve intestinal function during aging remains relatively sparse.

*Chrysosplenium axillare* Maxim., a Xizang medicinal plant, has been reported as a rich source of bioactive polymethoxy flavonoid glycosides, among which Chrysosplenoside I (CA1) and Chrysosplenoside A (CA2) were detected as the abundant ingredients [10, 11]. CA1, in particular, is a novel compound isolated from this plant. CAs 1 & 2 significantly improve acute intrahepatic cholestasis type liver injury in mice induced by alpha-naphthyl isothiocyanate (ANIT) [11]. In our preliminary experiments, CAs 1 & 2 showed the potential as the anti-aging agents. However, the ability of CAs 1 & 2 to regulate ISC function and exert anti-aging effects has not been fully explored, presenting an opportunity to advance the development of anti-aging therapeutics targeting the gut.

*Drosophila melanogaster*, with its well-characterized genetic toolkit and similarities to mammalian tissue biology, serves as an excellent model for studying ISC behavior and aging [12, 13]. The *Drosophila* midgut, with its diverse cell types and ISC-driven regeneration, mimics the complexity of mammalian intestines [14, 15]. Aging in *Drosophila* is marked by uncontrolled ISC proliferation, leading to disrupted homeostasis and impaired function, making it an ideal system for studying the effects of natural compounds on ISC aging [16]. Complementing *Drosophila* studies, the mouse intestinal organoid model has become a cornerstone for investigating mammalian intestinal aging, injury repair, and ISC regulatory mechanisms [3, 17, 18]. The organoid system, a recognized *in vitro* model, mirrors the functionality of stem cells *in vivo*. The organoid-forming capacity largely depends on the vitality of ISCs [17]. This model recapitulates the aging-associated decline in regenerative capacity, proliferation, and differentiation seen in the mammalian gut [3], providing a robust platform for evaluating the efficacy of novel natural compounds.

In this study, we leverage both *Drosophila* and murine intestinal organoid models to investigate the therapeutic potential of CAs 1 & 2 in ameliorating age-related ISC dysfunction. We demonstrate that CAs 1 & 2 prevent excessive ISC proliferation, maintain intestinal homeostasis, and extend lifespan in *Drosophila*, while also promoting the growth and budding of organoids derived from aged mice. Our findings reveal that the activation of PPARγ signaling and inhibition of EGFR signaling are central to the action of CAs 1 & 2, positioning these compounds as promising geroprotective agents for clinical consideration in addressing age-related intestinal decline.

## Results

### CAs 1 & 2 treatment attenuates aging-induced dysfunction of intestinal stem cells

**Figure 1.**
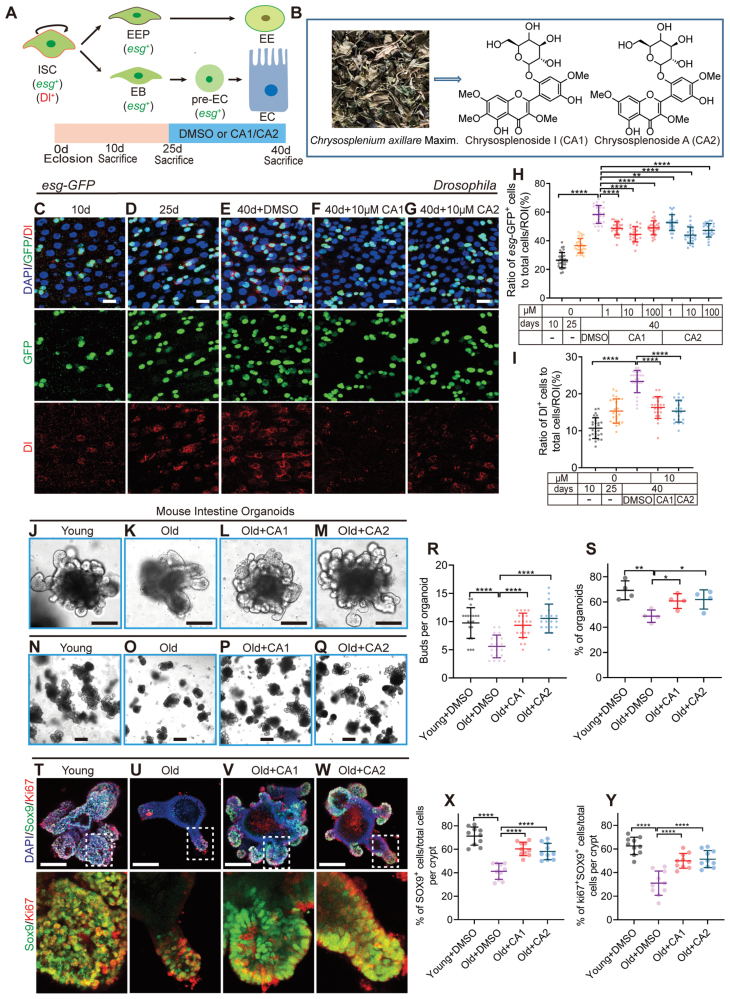
**CAs 1 & 2 treatment attenuates the dysfunction of ISCs induced by aging.** (A) A model of cell lineage and markers in *Drosophila* midgut epithelium and schematic diagram showing the process of feeding CAs 1 & 2 to *Drosophila*. EB, enteroblast; EEP, EE progenitor cell; EE, enteroendocrine cell; pre-EC, pre-enterocyte; EC, absorptive enterocyte. (B) Schematic structure of CAs 1 & 2, natural flavonoid glycosides from *Chrysosplenium axillare* Maxim. (C–G) Representative immunofluorescence images of midguts at 10-, 25-, and 40-days *Drosophila* (*esg-GFP/CyO*) with DMSO or 10 µM CAs 1 & 2 treatment and stained with DAPI (nuclei), *esg-*GFP (ISCs and progenitor cells marker), and Dl (ISCs marker). Scale bar: 10 μm. (H) The ratio of *esg*-GFP^*+*^ cells to total cells per region of interest (ROI) in the midguts of 10-, 25-day-old *Drosophila* (*esg-GFP/CyO*), and 40-day-old *Drosophila* (*esg-GFP/CyO*) fed with DMSO or three concentrations (1, 10, and 100 µM) of CAs 1 & 2. Each point represents a ROI in the midguts of *Drosophila*. (I) The ratio of Dl^+^ cells to total cells per ROI in the midguts of *Drosophila* in experiments (C–G). Each point represents a ROI in the midguts of *Drosophila*. (J–Q) Representative images of young mouse organoid and old mouse organoid treated or not with CAs 1 & 2. (J–M, scale bar: 100 μm; N–Q, scale bar: 400 μm) (R, S) Quantification of the buds (R) and the formation rates (S) of mouse organoid. Each point represents one organoid in (R) and one experimental repetition in (S). (T–W) Representative immunofluorescence images of young mouse organoid and old mouse organoid treated or not with CAs 1 & 2. Stained with DAPI, SOX9 (ISCs marker), and Ki67 (proliferating cell marker). Scale bar: 100 μm. (X, Y) The ratio of SOX9^+^ cells or Ki67^+^SOX9^+^ cells to total cells per crypt in experiments (T–W). Each point represents a crypt. Data information: Error bars represent standard deviation (SDs). Student’s *t* tests, *P < 0.05; **P < 0.01; ***P < 0.001; ****P < 0.0001

In the midguts of young *Drosophila*, ISCs play a pivotal role in maintaining gut homeostasis through their regulated proliferation and differentiation (Fig. 1A) [19]. However, in aged *Drosophila*, ISCs exhibit an elevated rate of proliferation, and their progeny, the enteroblasts (EBs), fail to differentiate into mature intestinal cells. This failure leads to an accumulation of *esg*^*+*^ cells (comprising both ISCs and EBs) within the midgut, while the count of fully differentiated intestinal cells declines [16]. To track and quantify *esg*^*+*^ cells in real time, we employed a reporter gene system (*esg*-*GFP*/*CyO*), which expresses green fluorescent protein (GFP) under the control of the *escargot* (*esg*) gene, thereby facilitating the monitoring of age-related ISC overproliferation in *Drosophila* midguts. This system also enabled us to screen for small molecule drugs that could mitigate age-related ISC dysfunction.

During our initial screening, two naturally occurring small molecules extracted from *Chrysosplenium axillare* Maxim., CAs 1 & 2 (Fig. 1B), were found to significantly curb the accumulation of *esg*^*+*^ cells in the midguts of aged *Drosophila*. To delve deeper into the efficacy of CAs 1 & 2 in alleviating age-related ISC dysfunction, we administered diets containing three different concentrations (1, 10, 100 μM) of CAs 1 & 2 to middle-aged (25-day-old) *Drosophila* for a period of 15 days (Fig. 1A). The results pointed to the 10 μM concentration of CAs 1 & 2 as the most effective in reducing the accumulation of *esg*^*+*^ cells in the midguts of aged *Drosophila* (Fig. 1C–H and S1A–G).

Further validation of CAs 1 & 2’s capabilities was obtained by using Delta (Dl) antibody staining to specifically label ISCs in aged *Drosophila*. The number of Dl^+^ ISCs was significantly lower in the group fed with 10 μM CAs 1 & 2 compared to the control group (Fig. 1C–G and I). In addition, we quantified the number of proliferating cells marked by pH3^*+*^ (phosphorylated-histone 3, a marker for proliferating cells), which indicated that CAs 1 & 2 supplementation markedly inhibited age-related ISC overproliferation in *Drosophila* (Fig. S1H–M). These findings suggest that CAs 1 & 2 supplementation can prevent both age-related ISC overproliferation in aged *Drosophila*.

In mammals, aged intestinal crypts exhibit a decrease in number, delayed proliferation, and increased cell apoptosis. Correspondingly, the proliferation and budding rates of intestinal organoids *in vitro* also decline [20]. To further assess the anti-aging effects of CAs 1 & 2, we measured the organoid formation frequency in young and aged mouse intestinal crypts. We treated organoids derived from both aged intestines with 10 μM CAs 1 & 2, and after 7 days of cultivation, we observed a significant improvement in the budding and formation rates of aged organoids compared to untreated young and aging controls (Fig. 1J, 1K, 1N, 1O, 1R, and 1S). However, the addition of CAs 1 & 2 led to an increase in the number and leaf/bud count of aged organoids, nearly restoring them to the levels observed in young organoid cultures (Fig. 1J–S). Thus, CAs 1 & 2 supplementation appears to rejuvenate the function of aged organoids to a state akin to that of young organoids.

To further substantiate the anti-aging effect of CAs 1 & 2, we utilized SOX9 and Ki67 to label ISCs and proliferating cells. The results showed a significant increase in the number of SOX9^*+*^ cells and Ki67^*+*^SOX9^*+*^ cells in each crypt of aged intestinal organoids treated with CAs 1 & 2 compared to untreated ones (Fig. 1T–Y). Taking all these data into consideration, we conclude that CAs 1 & 2 treatment can alleviate age-induced functional impairment of ISCs.

### CAs 1 & 2 protect intestinal function and mitigate oxidative stress and inflammation in aged Drosophila

**Figure 2.**
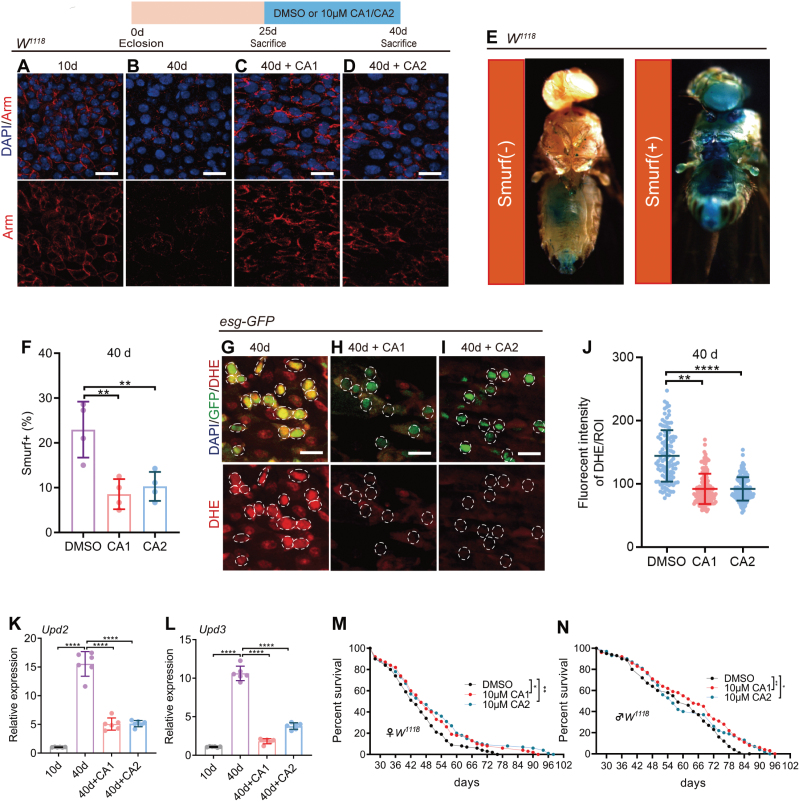
**CAs 1 & 2 against age-related intestinal dysfunction and attenuate ROS and inflammation in *Drosophila.*** (A–D) Representative immunofluorescence images of midguts of 10- and 40-day-old *Drosophila* (*W*^*1118*^) without CAs 1 & 2 supplementation and 40-day-old *Drosophila* (*W*^*1118*^) with 10 µM CAs 1 & 2 supplementation. Stained with DAPI, Arm (red; cellular connection marker). Scale bar: 10 μm. (E, F) Representative images (E) and quantification (F) of the percentage of the smurf *Drosophila* in 40-day-old *Drosophila* (*W*^*1118*^) with DMSO or 10 µM CAs 1 & 2 supplementation. Smurf (+) refers to a fly that exhibits the leakage of a blue dye from the gut into other tissues. Each group consisted of 15 *Drosophila*, and the experiments were independently conducted four times. Each point represents one experimental repetition in (F). (G–I) Representative immunofluorescence images of midguts of 40-day-old *Drosophila* (*esg-GFP/CyO*) with DMSO or 10 µM CAs 1 & 2 supplementation. Stained with DAPI, *esg-*GFP, DHE (ROS marker). Scale bar: 10 μm. (J) Quantification of DHE fluorecent intensity per ROI in the midguts of *Drosophila* in experiments (G–I). Each point represents a ROI in the midgut of *Drosophila*. (K, L) Relative mRNA fold changes of inflammatory-related genes with whole gut of 10-day *Drosophila* (*W*^*1118*^) and 40-day *Drosophila* (*W*^*1118*^) treated with DMSO or 10 µM CAs 1 & 2 supplementation. Each group had 15 *Drosophila* were conducted. Each point represents one experimental repetition. (M, N) Survival percentage of female *W*^*1118*^ *Drosophila* with DMSO or CA1 or CA2 supplementation starting from middle age (26-day-old). Each group included 100 *Drosophila*. Three independent experiments were conducted. Data information: Error bars represent SDs. Log-rank test was used for lifespan analysis. Student’s *t* tests, *P < 0.05; **P < 0.01; ***P < 0.001; ****P < 0.0001. Non-significance (ns) represents *P* > 0.05.

As *Drosophila* age, their intestinal functionality markedly diminishes, the intestinal barrier is damaged, inflammation ensues, and oxidative stress escalates, prompting immune activation [3]. Our study reveals that CAs 1 & 2 can suppress the overproliferation of ISCs in aged *Drosophila*, which is vital for preserving intestinal health. Consequently, we delved into whether CAs 1 & 2 could decelerate the deterioration of intestinal function in aging *Drosophila*. Initially, we employed Armadillo (Arm) staining and smurf assays to evaluate the integrity of the *Drosophila* intestinal epithelial barrier [21]. The findings suggest that CAs 1 & 2 supplementation mitigates the dysfunction of the intestinal barrier in aged *Drosophila* (Fig. 2A–F). To determine if CAs 1 & 2 could alleviate intestinal oxidative stress in aging *Drosophila*, we utilized the dihydroethidium (DHE) fluorescent probe to measure reactive oxygen species (ROS) levels. Our observations confirmed that CAs 1 & 2 supplementation notably diminishes ROS levels in aging *Drosophila* (Fig. 2G–J), showcasing CAs 1 & 2’s potential in reducing oxidative stress.

Moreover, we examined the influence of CAs 1 & 2 on the intestinal immune response during *Drosophila* aging. The aging process is characterized by a significant upsurge in the expression of inflammatory cytokines, such as the *IL-6*-like cytokines *upd2* and *upd3* in *Drosophila* [22]. Post CAs 1 & 2 treatment, there was a substantial reduction in the expression of these cytokines (Fig. 2K and 2L), signifying that CAs 1 & 2 effectively dampen the inflammatory response in aging *Drosophila*. Additionally, we probed whether CAs 1 & 2 supplementation could prolong the lifespan of *Drosophila*. The data revealed that CAs 1 & 2 extend the lifespan of both male and female *Drosophila* (Fig. 2M and 2N). To eliminate the variable of food intake on lifespan, a harmless blue dye was added to the food in the study. By measuring the intensity of the blue color in the *Drosophila*’ abdomens, the amount of food consumed was directly quantified, providing a reliable indicator of food intake [23]. Preliminary results showed that there was no significant difference in food consumption between *Drosophila* treated with CAs 1 & 2 and the untreated control group (Fig. S1N and S1O). This indicates that the observed anti-aging effects of CAs 1 & 2 are not due to a reduction in food consumption. In essence, CAs 1 & 2 are pivotal in thwarting age-related intestinal dysfunction, curtailing ROS levels, and quelling inflammatory responses in *Drosophila*, ultimately contributing to lifespan extension.

### CAs 1 & 2 curtail age-related ISC hyperproliferation via inhibiting EGFR pathway

**Figure 3.**
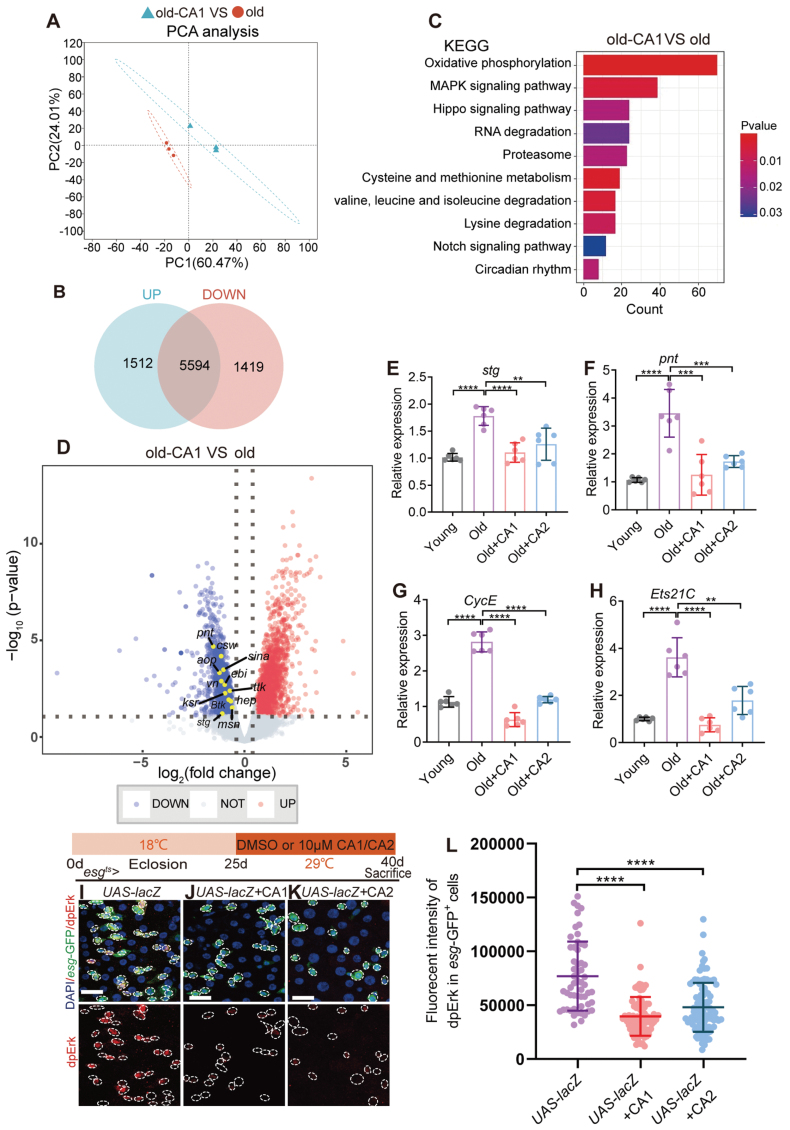
**CAs 1 & 2 prevent age-associated hyperproliferation of ISCs by inhibiting the EGFR signaling pathway.** (A) PCA of the gut in the old group (40 d) and old-CA1-treated *Drosophila*. (B) The differential gene expression of aging *Drosophila* with CA1 treatment to old group in Venn diagram. (C) KEGG pathway enrichment analysis of the enrichment pathways influenced by CA1. (D) Volcano plot shows differentially expressed genes in CA1-treated 40-day-old *Drosophila* compared to the control group. *pnt*, *msn*, *stg*, *ksr*, and others are EGFR-related genes. (E–H) Relative mRNA fold changes of the core target genes of the EGFR signaling pathway with midguts of 10-day (young) *Drosophila* (*W*^*1118*^) and 40-day (old) *Drosophila* (*W*^*1118*^) treated with DMSO or 10 µM CAs 1 & 2 supplementation. Each group had 15 *Drosophila* were conducted. Each point represents one experimental repetition. (I–K) Representative immunofluorescence images of midguts of 40-day-old *Drosophila* carrying *esg*^*ts*^*-Gal4*-driven *UAS-lacZ* (I), *UAS-lacZ* + CA1 (J), *UAS-lacZ* + CA2 (K), stained with DAPI, *esg*-GFP, dpErk (EGFR pathway activation marker). Scale bar: 10 μm. (L) Quantification of dpErk fluorescent intensity in *esg-*GFP^*+*^ cells in the midguts of *Drosophila* in experiments (I–K). Each point represents one *esg-*GFP^*+*^ cell in the midgut of *Drosophila*. Data information: Error bars represent SDs. Student’s *t* tests, *P < 0.05; **P < 0.01; ***P < 0.001; ****P < 0.0001. ns represents *P* > 0.05.

To unravel the underlying mechanisms of CAs 1 & 2, we embarked on RNA sequencing of the intestines from aged *Drosophila* with or without CA1 supplementation. Principal component analysis (PCA) disclosed a pronounced divergence between the old group and the old-CA1 group (Fig. 3A), signifying CA1’s substantial impact on the transcriptome of the aging *Drosophila* intestine. A Venn diagram illustrating the differential gene expression (Fig. 3B) indicated that the old-CA1 group exhibited 1419 genes with reduced expression and 1512 genes with increased expression relative to the old group, highlighting notable transcriptional changes. Further Kyoto Encyclopedia of Genes and Genomes (KEGG) pathway analysis (Fig. 3C) pinpointed the enrichment of several pathways influenced by CA1, including MAPK, Hippo, proteasome, and Notch, which are integral to cellular growth and division. These signaling pathways are closely linked to the epidermal growth factor receptor (EGFR) signaling pathway. EGFR is a recognized regulator of ISC proliferation and differentiation, becoming aberrantly active as *Drosophila* ages (Fig. S2A) [16]. Hence, we deduce that CA1 mitigates the abnormal proliferation of aging ISCs via the EGFR pathway.

To prove this hypothesis, we examined the expression of EGFR signaling pathway-associated genes through volcano plots. Furthermore, we found that CA1 can inhibit the key targets of EGFR such as *pnt*, *msn*, *stg*, *ksr,* and others (Fig. 3D). Subsequent RT-qPCR validation of EGFR pathway-related genes (*stg*, *pnt*, *CycE*, *Ets21C*) corroborated our findings, demonstrating that CA2, akin to CA1, inhibits the core targets of the EGFR signaling pathway (Fig. 3E–H). These outcomes collectively suggest that CAs 1 & 2 impede the EGFR signaling pathway. To further confirm CAs 1 & 2’s inhibitory influence on the EGFR pathway and its role in the abnormal ISC proliferation in aging *Drosophila*, we assessed the expression of dpErk (a marker of the EGFR pathway activation) in *esg*-GFP^*+*^ cells. The results indicated that CAs 1 & 2 supplementation markedly curtailed the activation of the EGFR signaling pathway in aging *Drosophila* (Fig. 3I–L), thereby substantiating the hypothesis that CAs 1 & 2 mitigate the excessive proliferation of ISCs by inhibiting the EGFR signaling pathway.

### CAs 1 & 2 as activators of the PPARγ signaling pathway

During our investigation, besides the EGFR pathway, RNA-seq results also showed a significant upregulation of PPARγ core target genes, including *pex5*, *pex10*, *pex19*, and *pex11* (Fig. S2B). This upsurge aligns with our existing literature that underscores the pivotal role of PPARγ target genes in modulating the proliferation and differentiation of ISCs [24]. Moreover, through volcano plot analysis, we have specifically highlighted the decreased expression of genes such as *srp, E2f1, InR, Pi3K92E, Acc, FASN1, Glut4EF*, and *upd3*. This phenomenon reveals pathways associated with negative regulation related to the PPARγ signaling pathway (Fig. S2B). Furthermore, studies have demonstrated that during aging, a majority of PPARγ target genes are downregulated, and the activation of PPARγ has been associated with mitigating metabolic dysfunction in aged mice, thereby prolonging their lifespan [25]. These insights suggest that the activation of PPARγ may be instrumental in rejuvenating the functionality of aging *Drosophila* ISCs.

**Figure 4.**
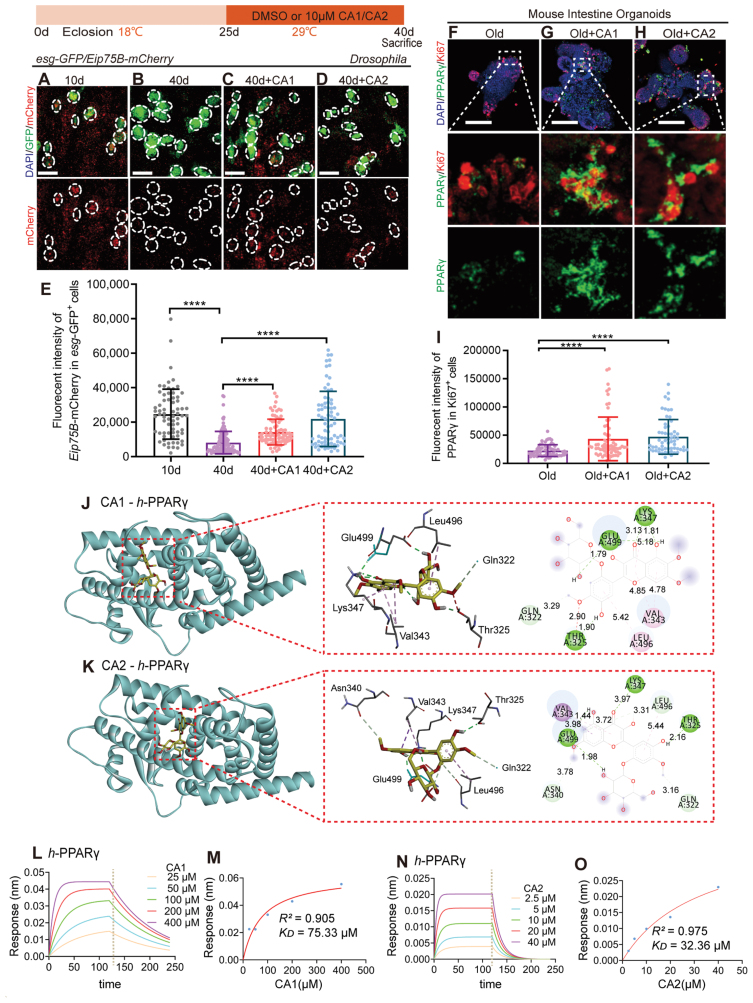
**CAs 1 & 2 activate the PPARγ signaling pathway.** (A–D) Representative immunofluorescence images of midguts of *Drosophila* carrying *esg-GFP*/*Eip75B-mCherry*, 10 day (A), 40 day (B), 40 day + CA1 (C), 40 day + CA2 (D). Stained with DAPI, *esg*-GFP, *Eip75B-*mCherry. (Scale bar: 10 μm). (E) Quantification of mCherry fluorecent intensity in *esg-*GFP^*+*^ cells in experiments (A–D). Each point represents one *esg-*GFP^*+*^ cell. (F–H) Representative immunofluorescence imags of old mouse organoid treated or not with CAs 1 & 2. Stained with DAPI, PPARγ and Ki67. (Scale bar: 100 μm). (I) Quantification of PPARγ fluorecent intensity in Ki67^*+*^ cells in experiments (F–H). Each point represents one Ki67^*+*^ cell. (J, K) Diagram of the molecular docking of CAs 1 & 2 with *h*-PPARγ, the binding energies for the targets docked into the CA1 is −3.88 kcal/mol (J) and CA2 is −2.75 kcal/mol (K). (L–O) Real-time kinetic binding sensorgrams and the equilibrium binding signal of different concentrations of CA1 increasing from 25 to 400 µM (L and M), CA2 increasing from 2.5 to 40 µM are shown (N and O). Response (nm) indicates the optical thickness on the NTA biosensor layer. Data information: Error bars represent SDs. Student’s *t* tests, *P < 0.05; **P < 0.01; ***P < 0.001; ****P < 0.0001

Given the documented interaction between flavonoids and PPARγ, we posited that CAs 1 & 2 might directly target PPARγ, thus revitalizing aging *Drosophila* ISCs. To explore this hypothesis, we employed *Drosophila* models expressing ISC markers (*esg*-GFP) and PPARγ (*Eip75B*-mCherry, *Drosophila*’s *PPARγ* homolog). Our observations revealed that in untreated aging *Drosophila*, the fluorescence intensity of Eip75B in *esg*-GFP^*+*^ cells was significantly diminished when compared to their younger counterparts. However, post-treatment with CAs 1 & 2, the fluorescence intensity of Eip75B in *esg*-GFP^*+*^ cells in aging *Drosophila* nearly matched that of the young (Fig. 4A–E). Complementary immunofluorescence staining of PPARγ protein in mouse intestinal organoids corroborated the findings from the *Drosophila* model (Fig. 4F–I), suggesting that CAs 1 & 2 can activate PPARγ across different species.

To substantiate the direct interaction between CAs 1 & 2 and PPARγ, we engaged in molecular docking studies that indicated a strong binding affinity between the two molecules (Fig. 4J and 4K). This was further validated using bio-layer interferometry (BLI) technology, which confirmed a direct and reversible interaction between CAs 1 & 2 and PPARγ. The interaction was characterized by a concentration-dependent increase in optical thickness (nm) on the sensor layer. The dissociation constants (*K*_D_) for CAs 1 & 2 and PPARγ were determined to be 75.33 μM and 32.36 μM, respectively (Fig. 4L–O), signifying a robust binding affinity. These findings collectively reinforce the notion that CAs 1 & 2 activate the PPARγ signaling pathway, providing a potential therapeutic avenue for age-related ISC dysfunction.

### CAs 1 & 2 mitigate age-related dysregulation of intestinal stem cell through PPARγ activation and EGFR inhibition

**Figure 5.**
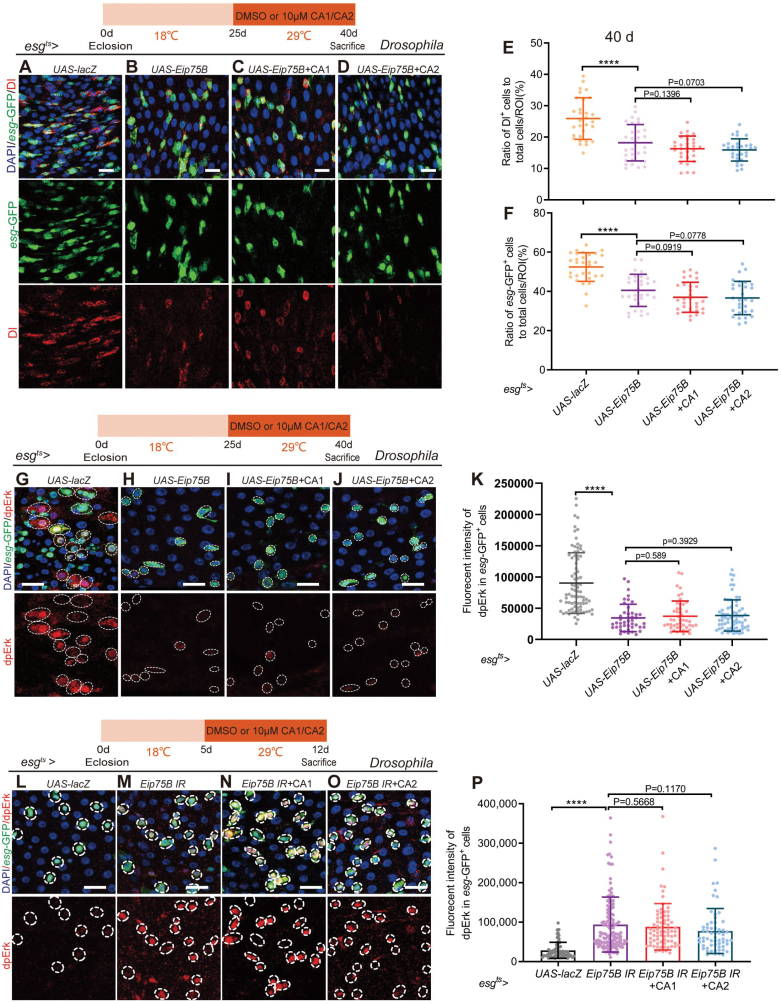
**CAs 1 & 2 alleviate age-related abnormal proliferation of ISCs via activating PPARγ to inhibit the EGFR signaling pathway.** (A–D) Representative immunofluorescence images of midguts of 40-day-old *Drosophila* carrying *esg*^*ts*^*-Gal4*-driven *UAS-lacZ* (A), *UAS-Eip75B* (B), *UAS-Eip75B* + CA1 (C), *UAS-Eip75B* + CA2 (D), stained with DAPI, *esg*-GFP, Dl. Scale bar: 10 μm. (E, F) The ratio of Dl^+^ cells (E) or *esg-*GFP^*+*^ cells (F) to total cells per ROI in the midguts of *Drosophila* in experiments (A–D). Each point represents an ROI in the midguts of *Drosophila*. (G–J) Representative immunofluorescence images of midguts of 40-day-old *Drosophila* carrying *esg*^*ts*^*-Gal4*-driven *UAS-lacZ* (G), *UAS-Eip75B* (H), *UAS-Eip75B* + CA1 (I), *UAS-Eip75B* + CA2 (J), stained with DAPI, *esg*-GFP, dpErk. Scale bar: 10 μm. (K) Quantification of dpErk fluorecent intensity in *esg-*GFP^*+*^ cells in experiments (G–J). Each point represents one *esg-*GFP^*+*^ cell. (L–O) Representative immunofluorescence images of midguts of 40-day-old *Drosophila* carrying *esg*^*ts*^*-Gal4*-driven *UAS-lacZ* (L), *Eip75B IR* (M), *Eip75B IR* + CA1 (N), *Eip75B IR* + CA2 (O), stained with DAPI , *esg*-GFP, dpErk. Scale bar: 10 μm. (P) Quantification of dpErk fluorescent intensity in *esg-*GFP^*+*^ cells in experiments (L–O). Each point represents one *esg-*GFP^*+*^ cell. Data information: Error bars represent SDs. Student’s *t* tests, *P < 0.05; **P < 0.01; ***P < 0.001; ****P < 0.0001. ns represents *P* > 0.05.

To further validate the above results, we utilized *esg*^*ts*^-*Gal4 Drosophila*, we drove the overexpression of *Eip75B*, the *Drosophila* homolog of *PPARγ*, to assess the impact of CAs 1 & 2 on ISCs. Our findings revealed that *Eip75B* overexpression markedly curtailed the aberrant proliferation of *esg*-GFP^*+*^ and Dl^+^ cells in aged *Drosophila* (Fig. 5A, 5B, 5E, and 5F), underscoring PPARγ’s pivotal role in modulating ISC proliferation. The addition of CAs 1 & 2 did not further diminish ISC counts, suggesting that the role of CAs 1 & 2 in regulating ISC proliferation may be based on PPARγ (Fig. 5A–F). Subsequently, we attenuated *Eip75B* expression (*Eip75B IR*) in young *esg*^*ts*^ *Drosophila* and examined the effects of CAs 1 & 2. The downregulation of *Eip75B* induced abnormal ISC proliferation, while CAs 1 & 2 supplementation failed to reverse this effect (Fig. S3A–F), reinforcing the notion that CAs 1 & 2’s activation of PPARγ signaling thwarts the aberrant proliferation of aging *Drosophila* ISCs. Moreover, to assess the functional similarity between PPARγ agonists and CAs 1 and 2, we added the PPARγ agonist pioglitazone to the organoids of aging mice. Using a similar experimental design as for CAs 1 and 2, we evaluated the impact of pioglitazone on the frequency of organoid formation in aging mice. The results indicate that pioglitazone significantly increased the budding and formation rates of aging organoids, demonstrating effects similar to those of CAs 1 and 2 (Fig. S3G–L).

Prevailing research suggests that PPARγ activation can suppress the EGFR signaling pathway [26], which is known to effectively regulate the abnormal proliferation of aging *Drosophila*. We hypothesize that CAs 1 & 2 decelerate ISC aging in *Drosophila* by activating PPARγ, thereby impeding the EGFR signaling pathway. To test this hypothesis, we induced *Eip75B* overexpression in *esg*^*ts*^ *Drosophila* and monitored dpErk expression in the ISCs of aging *Drosophila*, with and without CAs 1 & 2 treatment. The results demonstrated that *Eip75B* overexpression significantly lowered dpErk levels in ISCs, and CAs 1 & 2 treatment not reduced dpErk expression compared to the untreated cohort (Fig. 5G–K). Further investigation showed that *Eip75B* knockdown in young *Drosophila* led to a notable surge in dpErk expression in ISCs, which CAs 1 & 2 treatment did not ameliorate (Fig. 5L–P). These findings suggest that CAs 1 & 2 dampen the EGFR signaling pathway through PPARγ activation, thereby mitigating the abnormal proliferation of aging *Drosophila* ISCs. In conclusion, CAs 1 & 2, by engaging the PPARγ signaling pathway and inhibiting the EGFR signaling pathway, alleviate the abnormal proliferation of aging *Drosophila* ISCs.

## Discussion

**Figure 6.**
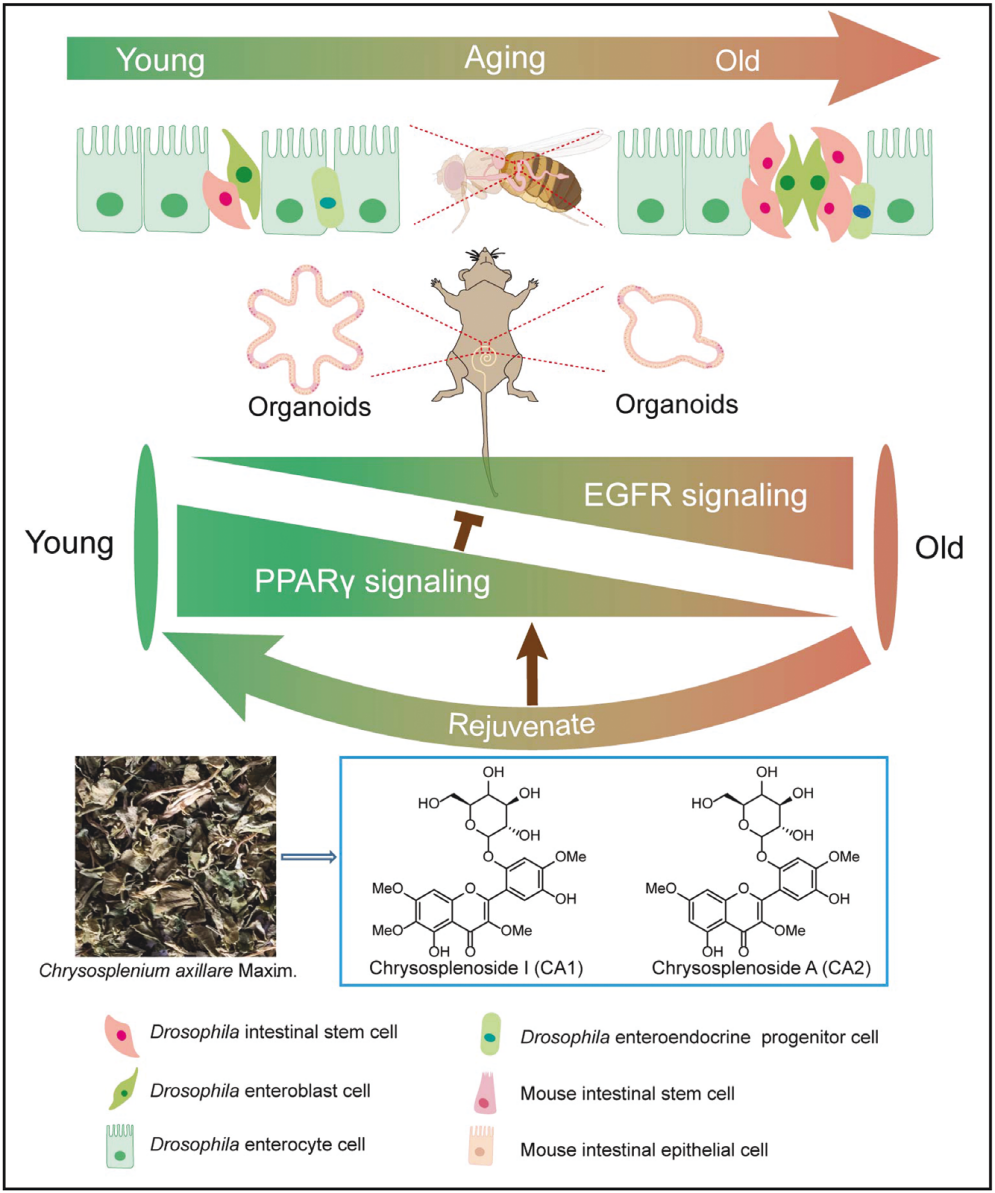
Graphical summary of CAs 1 & 2 rejuvenate ISC aging.

The quest for interventions that can mitigate the deleterious effects of aging on stem cell function is a critical frontier in biomedicine. Our study marks a significant advancement in this field. It uncovers a novel class of small molecular compounds, CAs 1 & 2, with potent anti-aging properties that regulate stem cell functionality. This achievement is made possible by achieving the inhibition of the EGFR pathway through the activation of PPARγ (Fig. 6). These findings not only deepen our comprehension of the molecular mechanisms underlying stem cell aging but also open up new avenues for the development of therapeutic strategies aimed at preserving tissue homeostasis and extending healthspan.

The progressive decline in the regenerative capacity of stem cells with age is a key contributor to tissue dysfunction and the onset of age-related pathologies [27]. Our findings suggest that CAs 1 & 2 can effectively mitigate this decline in ISCs by reducing oxidative stress and inflammation, which are common features of aging tissues. These effects are consistent with the well-documented anti-aging properties of flavonoids, which are known for their low toxicity and natural derivation [7, 28]. The capacity of CAs 1 & 2 to improve survival and stress tolerance further underscores their value as potential anti-aging agents.

The molecular mechanisms underlying the anti-aging effects of CAs 1 & 2 appear to be mediated through the modulation of the PPARγ signaling pathway. Given the established role of PPARγ in lipid metabolism [29], inflammation [30], and oxidative stress [31], its activation by CAs 1 & 2 offers a plausible explanation for the observed enhancement of ISC function and longevity. Moreover, the inhibition of the EGFR signaling pathway by CAs 1 & 2 through PPARγ activation adds another layer of complexity to their mode of action, providing a dual mechanism by which these compounds can exert their beneficial effects on ISCs.

Our use of the *Drosophila* intestine and mouse intestinal organoid models have proven to be highly effective in studying ISC function and aging [20, 32, 33]. These systems have facilitated rapid and cost-effective research, circumventing the challenges associated with longer reproductive cycles in mammalian models. Importantly, they have enabled us to validate the efficacy of CAs 1 & 2 in two distinct, yet complementary, model systems, thereby strengthening the case for their potential application in human medicine.

In summary, our research provides evidence that CAs 1 & 2 have anti-aging effects on ISC, mediated through the activation of the PPARγ signaling pathway and inhibition of the EGFR signaling pathway. These findings suggest that CAs 1 & 2 may be promising candidates for developing interventions to promote tissue regeneration in elderly patients and prevent age-related diseases.

## Research limitations

While our study provides a foundation for the therapeutic potential of CAs 1 & 2, further research is necessary to fully understand the molecular mechanisms through which these compounds act. It will be important to identify the downstream targets of PPARγ signaling that mediate the anti-aging effects observed in our study. Additionally, the long-term safety and efficacy of CAs 1 & 2, as well as their systemic impact across various tissues, remain to be explored.

## Methods

### Research ethics

The animal study was approved by the Experimental Animal Ethics Committee of West China Hospital of Sichuan University. It was conducted in accordance with local legislation and institutional guidelines. The ethics approval numbers are 20240425005 for mice and 20240425006 for *Drosophila*.

### Fly stocks and husbandry

The following *Drosophila* lines were used: *esg-GFP/CyO* line, *UAS-lacZ* line and *tub-Gal4* line (from Allan Spradling), *esg*^*ts*^*-Gal4* line (from Benjamin Ohlstein), *w*^*1118*^ line (BDSC# 3605), *UAS-EGFR*^*DN*^ line (BDSC# 5364), *UAS-EGFR*^*CA*^ line (BDSC# 9533), and *Eip75B IR* line (VDRC# 108399). The transgenic *Drosophila* lines *Eip75B-mCherry* and *UAS-Eip75B* were constructed in our laboratory. Genotypes for *Drosophila* in each figure panel are provided in Table S1. Female *Drosophila* were used for all experiments unless otherwise indicated.

Fly stocks were maintained on a standard cornmeal/agar medium (80 g sucrose, 50 g cornmeal, 20 g glucose, 18.75 g yeast, 5 g agar, and 30 mL propionic acid are dissolved in water for 1 L food), at 25°C, 65% humidity with a 12 h light:12 h dark cycle, except for those transgene expression using the *Gal4/Gal80*^*ts*^ system, which were restricted at 18°C and premised at 29°C.

### Crypt isolation and organoid culture

The mouse’s small intestine was excised and flushed with cold DPBS to remove fecal matter. It was then longitudinally opened with scissors, and villi were scraped off using glass slides. The cleaned intestine was cut into small segments using forceps, transferred to a 50 mL centrifuge tube, and washed with 15 mL of cold DPBS for 10–15 cycles until the supernatant appeared clear. After discarding the supernatant, the tissue was incubated with 25 mL of cold DPBS containing 5 mM EDTA on a shaker at 20 rpm at 8°C for 1.5 h. The solution was allowed to settle for 30 s before the supernatant was removed. The remaining supernatant was pipetted out with DPBS supplemented with 0.1% fetal bovine serum, and the mixture was filtered through a 70 µm strainer into a new 50 mL centrifuge tube, kept on ice. This step was repeated to collect 2**–**4 fractions.

From each fraction, 50 µL was sampled into a well of a 24-well plate to estimate crypt numbers under a microscope, selecting the fraction with the highest crypt concentration. This fraction was centrifuged at 290 *g* at 2°C–8°C for 5 min, the supernatant was discarded, and the pellet was kept. The pellet was resuspended in 10 mL of cold DPBS with 0.1% BSA, centrifuged at 200 *g* at 2°C–8°C for 3 min, and the supernatant was removed, leaving the crypts. The crypts were then resuspended in 10 mL of cold DMEM/F-12, and their density was estimated by counting the number in 10 µL aliquots (15 crypts per 10 µL equates to approximately 1500 crypts/mL). Crypt concentration was adjusted by centrifugation, mixed with matrix gel in a 1:1 ratio, and 50 µL was seeded per well in a 24-well plate. The plate was incubated at 37°C for 15 min to allow the matrix to solidify before adding 500 µL of mouse intestinal organoid medium containing dimethyl sulfoxide (DMSO)/CA1/CA2 to each well. The plate was incubated at 37°C and 5% CO_2_, with medium changes every 2**–**3 days. After 7 days, organoids were counted and collected for downstream processing.

### Immunofluorescence and microscopy for Drosophila midguts and mouse intestinal organoid

*Drosophila* midguts were dissected in 1× PBS and fixed at room temperature for 30 min in 4% paraformaldehyde (PFA) and heptane (for anti-dpErk immunostaining, guts were fixed in 8% paraformaldehyde for 50 min), then washed twice with methanol and three times with 0.1% Triton X-100 in PBS with shaking. Samples were incubated with primary antibody at 4°C overnight, the following primary antibodies were used: chicken anti-GFP, Abcam, 1:1000; mouse anti-Delta, DSHB, 1:50; rabbit anti-phosphoHistone H3 (Ser10), Millipore, 1:1000; mouse anti-Armadillo, DSHB, 1:50; rabbit anti-dpERK, Cell Signaling, 1:500; mouse anti-mCherry, Cell Signaling, 1:1000. After washing, guts were incubated with secondary antibodies (Alexa 488, 568 or 647, Invitrogen, 1:2000) and DAPI (Sigma, 1 µg/mL) for more than 2 h at room temperature with shaking. After washing, samples were mounted on glass slides for microscopy.

Aspirate the mouse intestinal organoid culture medium, and wash each well three times with 0.5 mL of PBS, for 5 min each time. The cells were fixed for half an hour at room temperature using 0.5 mL of 4% PFA solution. After fixation, the cells were washed three times with PBS, and placed on a shaker at room temperature for 5 min each time. Permeabilization was performed using 0.5 mL of PBS containing 0.1% Triton X-100 for half an hour at room temperature. The solution was removed, and the cells were incubated with the primary antibody at 4°C overnight, followed by three washes with 0.1% Triton X-100 (PBST). After the washes, the cells were incubated with the secondary antibody (Alexa 488 or 568, Invitrogen, 1:1000) and DAPI for 2 h at room temperature. Following this, the cells underwent three additional washes, and an anti-fluorescence quencher was added dropwise to each well. All of the antibodies were used according to the manufacturer’s instructions. The following commercial antibodies were used: anti-PPARγ (Proteintech, 16643-1-AP, 1:100); anti-SOX9 (Abways, CY5400, 1:400); anti-Ki67 (Servicebio, GB121141, 1:300).

All images were acquired on a Leica TCS-SP8 confocal microscope and processed with Leica Application Suite X, Adobe Illustrator, Photoshop, and ImageJ software.

### RT-qPCR

For each genotype, approximately 30 midguts were dissected, and total RNA was extracted using the RNA-easy Isolation Reagent (Vazyme, CAT# R701-01). Total RNA was used for cDNA synthesis with the *Evo* M-MLV RT Kit (Vazyme, CAT# P612-01). RT-qPCR was performed by mixing cDNA with ChamQ Universal SYBR qPCR Master Mix (Vazyme, CAT# Q712-02) and primers in 384-well plates. The relative expression of target genes was calculated by the 2^−ΔΔCT^ method, and *rp49* was used as the reference gene. For each experiment, at least three independent biological replications were used. Primers are listed in Table S2.

### CAs 1 & 2 treatment for Drosophila and mouse intestinal organoid

CAs 1 & 2 were extracted from *Chrysosplenium axillare* Maxim. of Xizang medicine (purity ≥ 98%). Initially, CAs 1 & 2 were dissolved in DMSO and then thoroughly mixed with standard food to achieve various concentrations (1, 10, 100 µM). Subsequently, the *Drosophila* were randomly gathered and evenly placed into vials containing either CAs 1 & 2 or DMSO (as a control) mixed food.

For the treatment of CAs 1 & 2 in mouse intestinal organoids, CAs 1 & 2 are initially dissolved in the organoid culture medium to a final concentration of 10 µM. Subsequently, administration is performed during the initial stage of organoid culture, followed by a 7-day observation period.

### Lifespan assays

For each group, 100 *Drosophila* of the same genetics were collected and separated equally into five vials, which contained the standard medium or medium supplemented with 10 µM CAs 1 & 2. The number of dead *Drosophila* was counted and the food was exchanged every 2 days. The assay was repeated as three independent experiments.

### DHE staining

DHE staining was performed to detect the ROS. The guts were dissected and incubated with 30 μM DHE (MKbio, #MX4812) on a shaker at room temperature for 10–20 min, then washed three times in PBS for 5 min each time. Samples were fixed in 4% PFA at room temperature for 30 min, after washing three times, samples were mounted on glass slides for microscopy immediately. The above operations were carried out in the dark.

### Smurf assay

The smurf assay was performed to assess the integrity of intestinal barrier function by measuring the distribution of a non-absorbable blue food dye (Spectrum Chemical Manufacturing Corp, #FD110) in *Drosophila* [34]. After being starved for 2 h, 20 *Drosophila* in each group were transferred into a medium with blue food dye (2.5%, *w*/*v*) for 12 h. If the blue dye is distributed in the body cavity outside the intestine, the intestinal barrier is incomplete, that is, Smurf (+) *Drosophila*. At least three independent biological replicates were performed.

### Blue food dye intake in Drosophila

Ten *Drosophila*, either female or male of 7-old-day, were subjected to a 24-h starvation period in vials filled with 1% agar in PBS. Subsequently, they were moved to vials containing 1% agar, 5% sucrose, and 2.5% blue food dye (Spectrum Chemical Manufacturing Corp, #FD110). Following a 15-min feeding session, 10 *Drosophila* were instantly frozen, then homogenized in 300 μL of PBS, and centrifuged for 25 min at 13,200 rpm. The optical density of the resulting supernatant was determined at 625 nm using a spectrophotometer.

### RNA-seq

RNA sequencing was performed using midguts supplemented with or without CA1. Total RNA was extracted using TRIzol Reagent (Magen). The library preparations were sequenced on Illumina Novaseq 6000 and 150 bp paired-end reads were generated. The RNA sequencing was performed by Shanghai Applied Protein Technology (China).

The raw sequencing data was initially filtered using Trimmomatic (version 0.39), and the de-duplicated consensus sequences were then mapped to *Drosophila melanogaster* BDGP6 using the STAR software (version 2.7.8). Read count extraction and normalization were carried out using featureCounts (Version 2.0.6), followed by the calculation of RPKMs. A *P*-value < 0.05 and fold-change > 1.5 were utilized to determine the statistical significance of gene expression differences. Analysis and KEGG enrichment analysis for differentially expressed genes were both conducted using R (v 4.3.1).

### Molecular docking

The CAs 1 & 2 compound’s 3D structure, sourced from the SDF file in the PubChem database, was converted to a mol2 file using Open Babel 3.1.1. Autodock MGL Tools 1.5.7 facilitated the addition of hydrogen bonds, root detection, and rotatable bond settings, before saving in PDBQT format [35]. Key target proteins’ 3D structures, retrieved from the PDB database, were processed to remove water molecules and inactive ligands using PyMOL software, then hydrogenated, charged, and exported in PDBQT format via AutoDockTools 1.5.7 [36]. Molecular docking utilized AutoDock Vina 1.1.2 [37], with visualization of outcomes in Discovery Studio 2016 Client [38].

### BLI analysis

Purchase human PPARγ protein with a His tag (HY-P7999, MCE, USA) and conduct a BLI analysis. Dissolve the PPARγ protein in PBS at a concentration of 50 mg/mL. Pre-wet the Nickel-nitrilotriacetic acid (NTA) biosensor with PBS and record the baseline. Then directly immobilize the PPARγ protein onto the NTA biosensor in a 96-well black F-bottom plate (655209, Greiner, Germany). Dilute CAs 1 & 2 in PBS containing 10% DMSO and 0.01% Tween 20 to the appropriate concentration, with a final volume of 300 μL per well. Add an equal volume of PBS containing 10% DMSO and 0.01% Tween 20 to the wells as a control. The experiment consists of four main steps: loading for 300 s, baseline for 60 s, association for 120 s, and dissociation for 120 s, repeated cyclically. Data collection and analysis are performed using the ForteBio Octet K2 system (Sartorius, Germany) with its data collection and analysis software (Octet® Analysis Studio 13.0.1.35, Sartorius, Germany).

### Statistical analysis

All statistical analyses were performed using GraphPad Prism 9.3 software. Statistical significance was displayed as means ± SD from at least three independent repeated experiments. Differences between groups were assessed with unpaired two-tailed Student’s *t* tests, Log-rank test was used for the comparison of lifespan assay.

## Supplementary Material

lnae025_suppl_Supplementary_Material

## Data Availability

The RNA-seq data that support the findings of this study have been deposited in the Sequence Read Archive (SRA) under BioProject ID PRJNA1059607.
