# Supplementary material for: Natural flavonoid glycosides Chrysosplenosides I & A rejuvenate intestinal stem cell aging via activation of PPARγ signaling
Source: Life Med. 2024 Jun 28;3(3):lnae025. doi: 10.1093/lifemedi/lnae025 (PMC11749787; doi:10.1093/lifemedi/lnae025)
Supplement: lnae025_suppl_Supplementary_Material [file lnae025_suppl_Supplementary_Material.docx]

**Natural flavonoid glycosides Chrysosplenosides I & A rejuvenate intestinal stem cell aging via activation of PPARγ signaling**

Jinbao Ye^1,#^, La Yan^1,#^, Yu Yuan^1,#^, Fang Fu^1^, Lu Yuan^2^, Xinxin Fan^1^, Juanyu Zhou^1^, Yuedan Zhu^1^, Xingzhu Liu^1^, Gang Ren^2,*^, Haiyang Chen^1,*^

^1^Laboratory of Metabolism and Aging Research, Frontiers Science Center for Disease-related Molecular Network, State Key Laboratory of Respiratory Health and Multimorbidity and National Clinical Research Center for Geriatrics, West China Hospital, Sichuan University, Chengdu 610041, China

^2^Research Center of Natural Resources of Chinese Medicinal Materials and Ethnic Medicine, Jiangxi University of Chinese Medicine, Nanchang 330004, China

^#^These authors contributed equally to this work.

^*^Correspondence: chenhy82@scu.edu.cn (H.C.), 20091005@jxutcm.edu.cn (G.R.)

**Supplemental figure legends**

**Figure S1. Related to Figs. 1 and 2.**

**
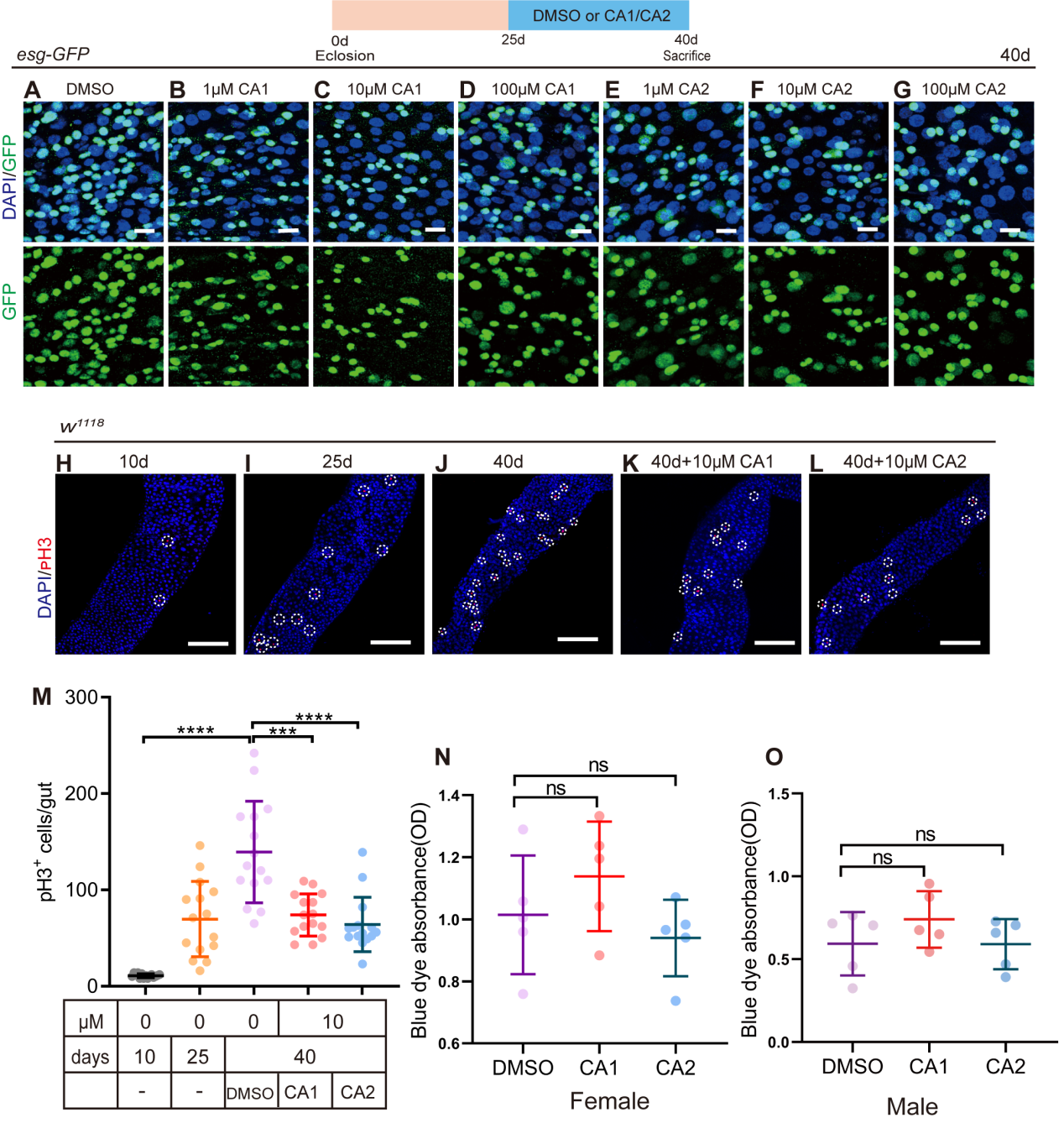
**

(A–G) Representative immunofluorescence images of midguts of 40-day-old *Drosophila* (*esg-GFP/CyO*) fed with DMSO or three concentrations (1, 10, and 100 µM) of CAs 1 & 2. Stained with DAPI (blue), *esg*-GFP (green). Scale bar: 10 μm.

(H–L) Representative immunofluorescence images of midguts at 10-, 25-, and 40-days Drosophila (*W^1118^*) with DMSO or 10 µM CAs 1 & 2 treatment and stained with DAPI (blue; nuclei), and pH3 (red; proliferating cells marker). Scale bar: 100 μm.

(M) Quantification of the pH3^+^ cells per gut in figure1 C-G. *Each point represents one gut.*

(N–O) Young female (N) and male (O) *Drosophila*, after a 24-hour period of fasting, were observed for their intake response to food marked with bright blue fluorescence. Each group consisted of 10 *Drosophila*. Each point represents one experimental repetition.

Data information: Error bars represent standard deviation (SDs). Student’s *t* tests, *, *p* < 0.05; **, *p* < 0.01; ***, *p* < 0.001; ****, *p* < 0.0001. non-significance (ns) represents *p* > 0.05.

**Figure S2. Related to Figs. 3 and 4.**

**
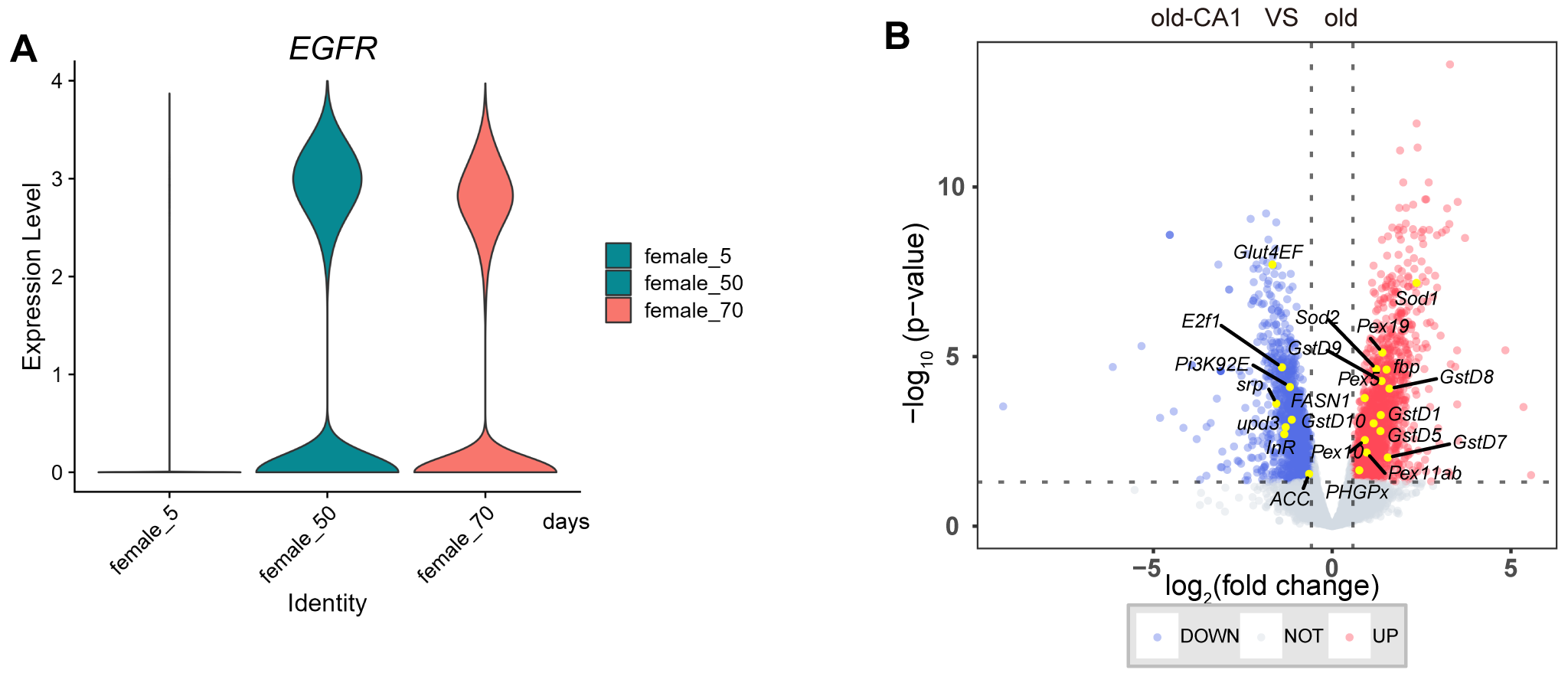
**

(A) Plot of age-related expression levels of *EGFR* in *Drosophila* ISCs. The data is from NCBI GEO under accession number GSE21866 [16].

(B) Volcano plot shows differentially expressed genes in CA1-treated 40-day-old *Drosophila* compared to the control group. Red dots indicate significantly regulated genes, blue dots indicate significantly downregulated genes, and gray dots indicate genes that are not significantly different.

**Figure S3. Related to Fig. 5.**

**
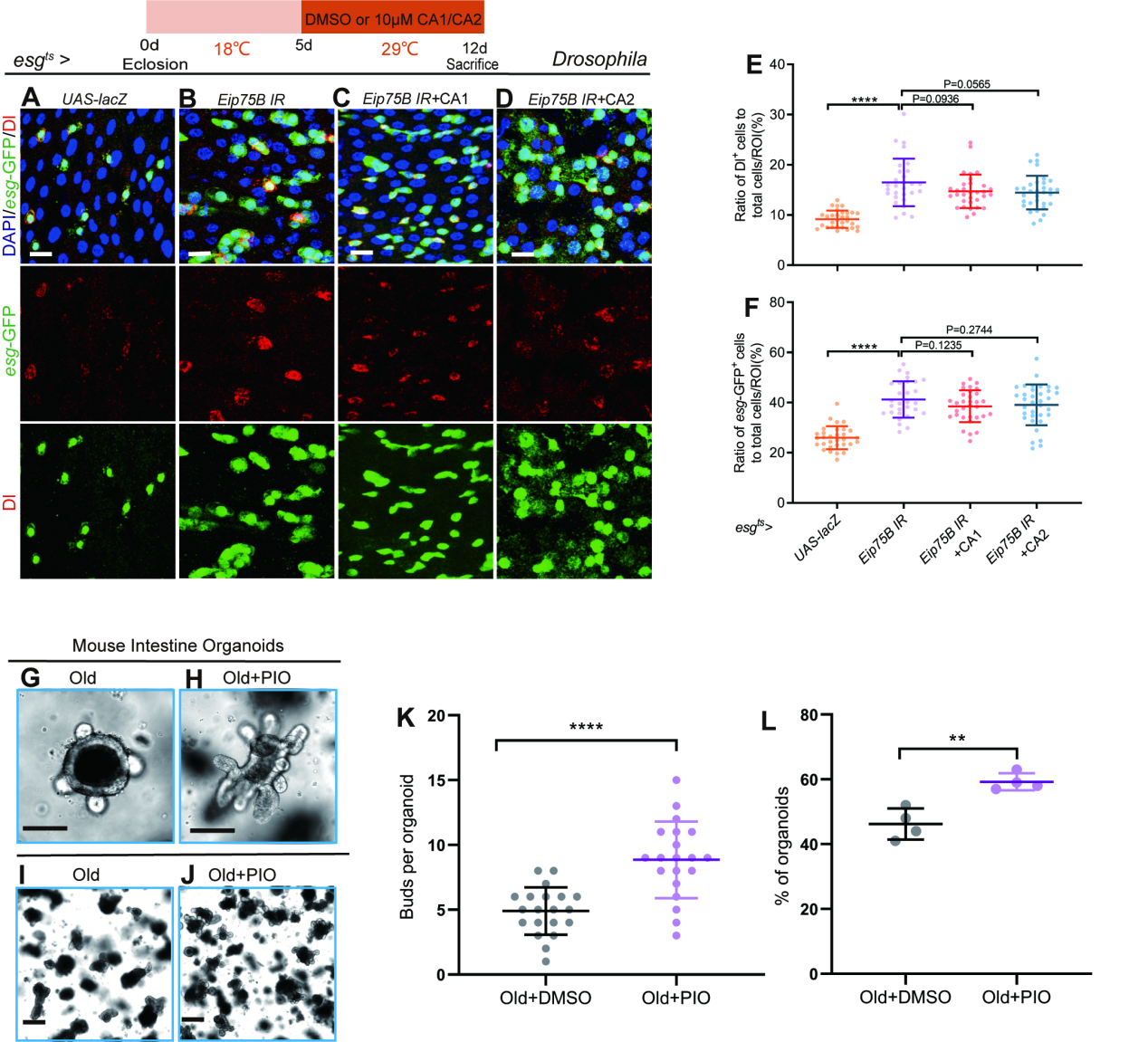
**

(A–D) Representative immunofluorescence images of midguts of 40-day-old *Drosophila* carrying *esg^ts^-Gal4*-driven *UAS-lacZ* (A), *Eip75B IR* (B), *Eip75B IR* + CA1 (C), *Eip75B IR* + CA2(D), stained with DAPI (blue), *esg*-GFP (green), Dl (red). Scale bar: 10 μm.

(E, F) The ratio of Dl^+^ or *esg-*GFP^+^ cells to total cells per ROI in the midguts of *Drosophila* in experiments (A–D). Each point represents a ROI in the midguts of *Drosophila*.

(G–J) Representative images of old mouse organoid treated or not with pioglitazone, PIO: pioglitazone. (G) and (H), scale bar: 100 μm; (I) and (J), scale bar: 400 μm.

(K, L) Quantification of the buds (K) and the formation rates (L) of mouse organoid. Each point represents one organoid in (K) and one experimental repetition in (L).

Data information: Error bars represent SDs. Student’s *t* tests, *, *p* < 0.05; **, *p* < 0.01; ***, *p* < 0.001; ****, *p* < 0.0001. ns represents *p* > 0.05.

| **Table S1 The list of full *Drosophila* genotypes** | |
| --- | --- |
| **Figure 1** | |
| **C**–**G** | *w^−^; esg-GFP/CyO; +/+* |
| **Figure 2** | |
| **A**–**E** | *w^−^; +/+; +/+* |
| **G**–**I** | *w^−^; esg-GFP/CyO; +/+* |
| **Figure 3** | |
| **I**–**K** | *w^−^; esg-Gal4, UAS-GFP, tub-Gal80^ts^/UAS-lacZ; +/+* |
| **Figure 4** | |
|  | *w^−^; esg-GFP/Eip75B-mCherry; +/+* |
| **Figure 5** | |
| **A, G, L** | *w^−^; esg-Gal4, UAS-GFP, tub-Gal80^ts^/UAS-lacZ; +/+* |
| **B**–**D, H**–**J** | *w^−^; esg-Gal4, UAS-GFP, tub-Gal80^ts^/+; UAS-Eip75B /+* |
| **M**–**O** | *w^−^; esg-Gal4, UAS-GFP, tub-Gal80^ts^/Eip75B IR; +/+* |
| **Figure S1** | |
| **A**–**G** | *w^−^; esg-GFP/CyO; +/+* |
| **H**–**L, N**–**O** | *w^−^; +/+; +/+* |
| **Figure S3** | |
| **A**–**D** | *w^−^; esg-Gal4, UAS-GFP, tub-Gal80^ts^/Eip75B IR; +/+* |

| **Table S2 The list of the primer sequences.** | |
| --- | --- |
| **Target** | **Sequence** |
| *upd2* | F: 5’- CGGAACATCACGATGAGCGAAT -3’ R: 5’- TCGGCAGGAACTTGTACTCG -3’ |
| *upd3* | F: 5’- ATCCCACCAATCCCCTGAAG -3’ R: 5’- AGATTGCAG GTGTTCTCCCA -3’ |
| *stg* | F: 5’- GAGCTGATGGGTCTGCTCTC -3’ R: 5’- ATGTGGAGGACAGGCTGTTG -3’ |
| *pnt* | F: 5’- ACGCCCTATGATGCTCAATC -3’ R: 5’- TATCCAGACCCAAGGTGCTC -3’ |
| *CycE* | F: 5’- ACAAATTTGGCCTGGGACTA -3’ R: 5’- GGCCATAAGCACTTCGTC -3’ |
| *Ets21C* | F: 5’- CCGGGCACTCAGGTACTACT -3’ R: 5’- CATACTGGAGGCCGGATCT -3’ |
| *Rp49* | F: 5’- ATCGGTTACGGATCGAACAAGC -3’ R: 5’- GTAAACGCGGTTCTGCATGAGC -3’ |
